# Preclinical characterization of AMPA receptor potentiator TAK‐137 as a therapeutic drug for schizophrenia

**DOI:** 10.1002/prp2.479

**Published:** 2019-05-09

**Authors:** Maiko Tanaka, Akiyoshi Kunugi, Atsushi Suzuki, Noriko Suzuki, Motohisa Suzuki, Haruhide Kimura

**Affiliations:** ^1^ Neuroscience Drug Discovery Unit, Research Takeda Pharmaceutical Company Limited Fujisawa Japan

**Keywords:** AMPA, AMPA receptor potentiator, schizophrenia

## Abstract

The downregulation of the glutamate system may be involved in positive, negative, and cognitive symptoms of schizophrenia. Through enhanced glutamate signaling, the activation of the α‐amino‐3‐hydroxy‐5‐methyl‐4‐isoxazole‐propionic acid (AMPA) receptor, an ionotropic glutamate receptor, could be a new therapeutic strategy for schizophrenia. TAK‐137 is a novel AMPA receptor potentiator with minimal agonistic activity; in this study, we used rodents and nonhuman primates to assess its potential as a drug for schizophrenia. At 10 mg kg^−1^ p.o., TAK‐137 partially inhibited methamphetamine‐induced hyperlocomotion in rats, and at 3, 10, and 30 mg kg^−1^ p.o., TAK‐137 partially inhibited MK‐801‐induced hyperlocomotion in mice, suggesting weak effects on the positive symptoms of schizophrenia. At 0.1 and 0.3 mg kg^−1^ p.o., TAK‐137 significantly ameliorated MK‐801‐induced deficits in the social interaction of rats, demonstrating potential improvement of impaired social functioning, which is a negative symptom of schizophrenia. The effects of TAK‐137 were evaluated on multiple cognitive domains—attention, working memory, and cognitive flexibility. TAK‐137 enhanced attention in the five‐choice serial reaction time task in rats at 0.2 mg kg^−1^ p.o., and improved working memory both in rats and monkeys: 0.2 and 0.6 mg kg^−1^ p.o. ameliorated MK‐801‐induced deficits in the radial arm maze test in rats, and 0.1 mg kg^−1^ p.o. improved the performance of ketamine‐treated monkeys in the delayed matching‐to‐sample task. At 0.1 and 1 mg kg^−1^ p.o., TAK‐137 improved the cognitive flexibility of subchronic phencyclidine‐treated rats in the reversal learning test. Thus, TAK‐137‐type AMPA receptor potentiators with low intrinsic activity may offer new therapies for schizophrenia.

**Abbreviations**
5‐CSRTT: five‐choice serial reaction time task
ADHD: attention deficit hyperactivity disorder
AMPA: (±)‐α‐amino‐3‐hydroxy‐5‐methylisoxazole‐4‐proprionic acid
D2R: dopamine D_2_ receptor
DMTS: delayed matching‐to‐sample
GlyT1: lycine transporter type 1
ITI: intertrial interval
METH: methamphetamine
NMDA: *N*‐methyl‐_D_‐aspartate
PCP: phencyclidine
RAM: radial arm maze
SI: social interaction
TAK‐137: 9‐(4‐phenoxyphenyl)‐3,4‐dihydropyrido[2,1‐c][1,2,4]thiadiazine 2,2‐dioxide

## 1 INTRODUCTION

Schizophrenia consists of a spectrum of symptoms: positive symptoms (hallucinations and delusions), negative symptoms (blunted affect and deficits in social functioning), and cognitive symptoms (deficits in attention, working memory, and cognitive flexibility).^1^, ^2^ The hyperdopamine hypothesis postulates that excessive activation of dopaminergic neurons in the subcortical regions of the brain is deeply involved in the pathophysiology of the positive symptoms of schizophrenia.^3^ Current antipsychotics, based on dopamine D2 receptor (D2R) antagonism, are effective against the positive symptoms of schizophrenia; however, their efficacy against the negative and cognitive symptoms is limited.^3^, ^4^ In addition, their side effects such as extrapyramidal symptoms and metabolic changes, limit their clinical application.^5^ Owing to the limitations of the efficacy and safety of the current medications, there is a large unmet need for novel therapeutic strategies for schizophrenia.

As postulated by the hypoglutamate hypothesis, the downregulation of glutamate signaling, especially the dysfunction of the *N*‐methyl‐D‐aspartate (NMDA) receptor in the medial prefrontal cortex plays an important role in schizophrenia.^6^, ^7^, ^8^ NMDA receptor antagonists, such as phencyclidine (PCP) and ketamine, induce not only psychotic symptoms, but also negative and cognitive symptoms in healthy subjects.^9^, ^10^, ^11^ Thus, the activation of the NMDA receptor may offer a potential therapeutic approach against schizophrenia. Bitopertin (RG1678), a glycine transporter type 1 (GlyT1) inhibitor, which can activate the NMDA receptor by increasing the level of the NMDA receptor co‐agonist (glycine),^12^ improved the negative symptoms of schizophrenia.^13^ Moreover, glycine or D‐serine—agonists at the glycine site of the NMDA receptor, significantly improved the negative symptoms of schizophrenia.^14^, ^15^ However, strategies that directly activate NMDA function have not been successful, possibly owing to the excitotoxic side effects 16, 17 or the desensitization of the NMDA receptors.^18^

The α‐amino‐3‐hydroxy‐5‐methyl‐4‐isoxazole‐propionic acid (AMPA) receptor is an ionotropic glutamate receptor that plays a key role in learning and memory.^19^ Glutamate receptor subunit 1‐knockout mice exhibited cognitive impairment, hyperdopaminergia, and psychosis‐like behaviors.^20^ The AMPA receptor is involved in the regulation of NMDA receptor activation, which induces ion influx into cells, triggering the release of channel‐blocking magnesium ion from the NMDA receptor. This results in the activation of NMDA receptor signaling through an increase in NMDA receptor‐mediated calcium influx.^21^ Therefore, the potentiation of the AMPA receptor is expected to offer a new therapeutic strategy for schizophrenia through the enhancement of glutamate signaling.

We recently discovered a novel AMPA receptor potentiator—TAK‐137 (9‐(4‐phenoxyphenyl)‐3,4‐dihydropyrido[2,1‐c][1,2,4]thiadiazine 2,2‐dioxide), which presented lower risks of a bell‐shaped dose response and seizure owing to its low agonistic activity.^22^, ^23^ TAK‐137 enhanced cognitive function in naive rats and nonhuman primates. In this study, we characterized TAK‐137 as a therapeutic drug for schizophrenia by using behavioral test batteries for the positive, negative, and cognitive symptoms of schizophrenia in rodents and nonhuman primates.

Methamphetamine (METH) enhances dopamine release and activates dopaminergic neurons in the subcortical regions of the brain, which causes psychotic symptoms that resemble the positive symptoms of schizophrenia.^24^ Based on hyperdopamine and hypoglutamate hypotheses for psychosis, METH and MK‐801 (an NMDA receptor antagonist) were used, respectively. As for cognitive functions, we evaluated the effects of TAK‐137 on multiple cognitive domains associated with schizophrenia, such as attention, working memory, and cognitive flexibility, using behavioral test batteries in rats and monkeys.^2^, ^25^ The data suggest that TAK‐137‐type AMPA receptor potentiators with low intrinsic activity may be a promising treatment for the multiple symptoms of schizophrenia, especially for negative and cognitive symptoms.

## 2 MATERIALS AND METHODS

### 2.1 Drug administration

TAK‐137 was synthesized by Takeda Pharmaceutical Company Limited (Fujisawa, Japan) and suspended in 0.5% (w/v) methylcellulose in distilled water; oral administration (p.o.) was conducted at a volume of 2 mL kg^−1^ in rats, 20 mL kg^−1^ in mice, and 5 mL kg^−1^ in monkeys. A solution of 0.5% methylcellulose was administered as the vehicle control. Methamphetamine hydrochloride (METH, Sumitomo Dainippon Pharma, Osaka, Japan) at 0.5 mg kg^−1^ and (+)‐MK‐801 hydrogen maleate (MK‐801, Sigma‐Aldrich St Louis, MO) at 0.08 or 0.1 mg kg^−1^ were dissolved in 0.9% saline and subcutaneously administered (s.c.) to rats at a volume of 2 mL kg^−1^. Phencyclidine hydrochloride (PCP, Sigma‐Aldrich, Poole, UK) (2 mg kg^−1^) was dissolved in 0.9% saline and administered intraperitoneally (i.p.) to rats at a volume of 1 mL kg^−1^ twice per day for 7 days. Ketamine hydrochloride (KETALAR^Ⓡ^, Daiichi Sankyo Propharma Co. Ltd, Tokyo, Japan) was dissolved in 0.9% saline and administered intramuscularly (i.m.) to monkeys at a volume of 5 mL kg^−1^.

### 2.2 Animals

Animal species and strains were selected based on the previous publications for each experiment. Male Sprague‐Dawley (SD) rats were purchased from Charles River Laboratories, Japan (Yokohama, Japan) for the measurements of locomotion (7‐week‐old rats).^26^ Male ICR mice and Wistar rats were purchased from CLEA Japan Inc. (Tokyo, Japan) for the measurement of locomotion (6‐week‐old mice)^26^ and the social interaction (SI) test (6‐week‐old rats), respectively. Male Long‐Evans rats were purchased from Japan SLC, Inc. (Hamamatsu, Japan) for the five‐choice serial reaction time task (5‐CSRTT) (6‐week‐old rats) and the radial arm maze (RAM) test (8‐week‐old rats).^27^ Female Lister Hooded rats (21 days postnatal) were supplied by Harlan Laboratories UK, Ltd. (Bicester, UK) for the reversal learning test.^28^ Female rats were used for reversal learning because females are highly sensitive to PCP 29 and showed more robust performance in certain cognitive tasks compared with male rats.^30^, ^31^ Rats and mice were housed in groups of four or five per cage in a light‐controlled room (12‐hour light/dark cycle; lights on at 7:00 AM) with free access to food and water. The room temperature and humidity were 20°C‐25°C and 40%‐60%, respectively, and the animals were given a minimum acclimation period of 1 week prior to the experiment. The animals were randomly assigned to the vehicle‐ or compound‐treated groups.

The delayed matching‐to‐sample (DMTS) task was evaluated in 4‐6‐year‐old male cynomolgus monkeys (*Macaca fascicularis*) weighing 4‐6 kg (Keari Co. Ltd., Osaka, Japan). The monkeys were housed individually in cages stored at a room temperature of (24 ± 1)°C and a humidity of (55 ± 15) %, with a 12‐hour light/dark cycle (lights on at 7:00 am). The monkeys were fed a complete, nutritionally balanced diet with fruit once daily (approximately 3:00‐4:00 pm) and water was available ad libitum. All monkeys were housed and handled in strict accordance with good animal practice under the supervision of veterinarians. They received environmental enrichment and were monitored for evidence of disease and changes in attitude, appetite, or behavior suggestive of illness. The care and use of the animals, and the experimental protocols used in this research except the reversal learning test, were approved by the Experimental Animal Care and Use Committee of Takeda Pharmaceutical Company Limited. The reversal learning test was carried out at b‐neuro^™^ (University of Manchester, UK) in accordance with the Animals Scientific Procedures Act, UK, and was approved by the Ethical Review Panel of the University of Bradford.

### 2.3 Locomotion measurement in rats

Locomotion was automatically evaluated using an infrared sensor system (SuperMex, Muromachi Kikai, Tokyo, Japan). The infrared sensor was placed on the center of the cover on the cage with a hole to detect locomotion. Each rat was placed in a Plexiglas covered cage (38 × 25 × 32 cm) for more than 2 hours before the experiment was started to allow acclimation to the experimental environment. Food and water were available ad libitum. After acclimation, TAK‐137 or the corresponding vehicle was administered p.o. 4 hours prior to METH administration (0.5 mg kg^−1^, s.c.). The numbers of animals used were as follows—vehicle/vehicle: 6, vehicle/METH: 12, TAK‐137 (0.1 mg kg^−1^)/METH: 11, TAK‐137 (1 mg kg^−1^)/METH: 13, and TAK‐137 (10 mg kg^−1^)/METH: 13. The data were collected as an accumulation of the counts of infrared sensors in each 3 minutes block after METH administration and are presented as the mean ± standard error of the mean (SEM) of the changes over time and the accumulation of activity counts for 120 minutes after METH administration. All the data were stored in a personal computer and analyzed by appropriate software (Comp ACT AMS, Muromachi Kikai, Tokyo, Japan).

### 2.4 Locomotion measurement in mice

Locomotion was automatically counted with an infrared sensor automated activity monitoring system. Each of the 36 test cages (30 × 40 × 20 cm) was equipped with a pyroelectric infrared sensor. Each mouse was allowed to acclimate to the test cage for more than 2 hours before the experiment was started. After acclimation, TAK‐137 or the corresponding vehicle was administered p.o. 2 hours before the s.c. administration of MK‐801 (Sigma‐Aldrich St Louis, MO) at 0.3 mg kg^−1^ in 0.9% saline or the corresponding vehicle. The numbers of animals used were as follows—vehicle/vehicle: 6, vehicle/MK‐801: 13, TAK‐137 (1 mg kg^−1^)/MK‐801: 14, TAK‐137 (3 mg kg^−1^)/MK‐801: 13, TAK‐137 (10 mg kg^−1^)/MK‐801: 16, and TAK‐137 (30 mg kg^−1^)/MK‐801: 15. The data were collected as accumulation of counts of infrared sensors in each 3 minutes block after MK‐801 administration and presented as the mean ± SEM of the changes over time and the accumulation of activity counts for 120 minutes after MK‐801 administration.

### 2.5 SI test

The experiment was performed as described previously 32 with some modifications. One day before the experiment, the rats were placed in the experimental room for 2 hours and orally administered distilled water for acclimation. On the day of the experiment, the rats were again placed in the experimental room for 2 hours for acclimation. After acclimation, TAK‐137 (0.1 or 0.3 mg kg^−1^) or the corresponding vehicle was administered p.o., followed by s.c. injection of MK‐801 (0.1 mg kg^−1^) or the corresponding vehicle at 4 hours before testing (n = 14 per group). Two rats in the same drug treatment group, from the different home cages but with a body weight difference of less than 15 g were placed diagonally opposite in a test box (60 × 60 × 60 cm, 35‐40 lux). The SI between two rats was determined from the total time spent participating in social behaviors such as sniffing, genital investigation, chasing, and fighting. The interaction time within a 10 minutes test period was measured by a researcher through the monitoring of the CCD camera viewing the test box under blind condition. Between each session, the test box was cleaned with 10% ethanol. In addition to the interaction time, the locomotor activity of each animal was measured by the tracking system in the same software (TopScan, CleverSys Inc., Reston, VA). The data are presented as the mean ± SEM of the interaction time and distance moved during the test.

### 2.6 5‐CSRTT

The experiment consisted of two sessions: training and testing. The training session started with 7‐week‐old male Long‐Evans rats, and 13 rats were used for the test at 12‐13 months of age. These rats were also used to assess the effects of drugs other than TAK‐137 on 5‐CSRTT performance between ages 6 and 12 months. They had a 2‐week washout period prior to the start of the study with TAK‐137. From the training session, food was restricted to 80%‐85% of the animals’ free‐feeding body weight, throughout the experimental period. The training and testing were conducted by using four operant chambers enclosed in sound‐attenuating boxes (Med Associates Inc., St Albans, VT). Each chamber contained a curved wall with five contiguous apertures. Food pellets were supplied automatically into a magazine located in the opposite wall of the five contiguous apertures in the chamber, and a photocell beam was used to detect head entries into the magazine. In the training session, a pellet was delivered into the magazine at the start of each session to initiate the first trial. After a 5 seconds intertrial interval (ITI), a light stimulus was presented in one of the 5 apertures, followed by a 5 seconds limited hold in the absence of light stimulus. The duration of the light stimuli was set to 5 seconds and gradually decreased during training to 2 seconds. The rats were required to nose‐poke into the illuminated aperture. The correct responses (nose‐poke responses in the illuminated aperture during a light stimulus and limited hold) resulted in the delivery of a food pellet into the magazine, with sound and light signals above the magazine occurring for 2 seconds. Rats with incorrect responses (nose‐poke responses in nonilluminated apertures), omissions (failure to respond during the limited hold), and premature responses (responses occurring prior to the presentation of the stimulus) were punished by a 5 seconds time‐out period, with extinction of the house light and no delivery of food. Each session lasted for 35 minutes or until 100 trials were completed. From a group of 15 trained rats, 13 rats that achieved the performance criteria (>75% correct responses and <20 omissions) over three consecutive days were used for the experiment. In the testing session, the duration of light stimuli on the aperture was set to 0.5 seconds. Vehicle or 0.2 mg kg^−1^ TAK‐137 was orally administered to the rats 4 hours prior to testing in a crossover fashion, with a washout period of 1 week. The data are indicated as the mean ± SEM of the number of correct responses, omissions, and premature responses.

### 2.7 RAM test

The experiment was performed as previously described^33^, ^34^ with a minor modification. The dimensions of each arm were 50 cm × 10 cm × 40 cm (length × width × height); the maze was elevated 50 cm above the floor. After a 24‐hour fast, the rats’ food intake was restricted to 80%‐85% of the free‐feeding body weight on the first day of exposure to the maze and throughout the experimental period. The RAM test consists of two sessions: training and testing. In the training session, rats were acclimated to the maze and then trained. Reinforcement consisted of 3 45 mg food pellets in a food cup. On the first day of acclimation to the maze, reinforcements were placed near the entrance and at the mid‐point of each arm. Three rats were placed on the maze at one time and allowed to explore and consume the pellets for 8 minutes. On the second day of acclimation, each rat was placed on the maze and allowed 5 minutes to consume the pellets in the food cups placed at the mid‐point and at the end of each arm. From the third day, reinforcements were placed in a food cup at the end of each arm. The rats were well‐trained to collect the pellets placed on the edge of each arm. The learning criterion for the testing session was defined as 2 errors or fewer for 2 consecutive days. From a group of 50 rats which were trained for 15 days, 36 rats that achieved the performance criterion were used for the experiment. On the previous day of the testing session, the baseline level of performance of the rats was assessed to select the rats that would complete the collection of all the pellets placed in the 8 arms with 2 errors or fewer. In the testing session, each rat was placed on the maze facing away from the researcher and facing the fixed arm at the start of the trial. The entry of rats into each arm was recorded in sequence. The rats were allowed to explore until all the pellets in the 8 arms were consumed, or 5 minutes had elapsed; entry into an arm previously chosen was counted as an error. If an animal left some of the 8 arms unexplored during the 5 minutes session, the number of unexplored arms was also counted as an error. TAK‐137 (0.2 and 0.6 mg kg^−1^, p.o.) or corresponding vehicle was administered 4 hours before the testing session. MK‐801 (0.08 mg kg^−1^, s.c., as a salt) or saline was administered 30 minutes before the testing session. The numbers of animals used were as follows— n = 7 in the vehicle/vehicle‐treated group, n = 9 in the vehicle/MK‐801‐treated group, n = 11 in the TAK‐137 (0.2 mg kg^−1^)/MK‐801‐treated group, and n = 9 in the TAK‐137 (0.6 mg kg^−1^)/MK‐801‐treated group. The data are presented as the mean ± SEM of errors in the testing session.

### 2.8 DMTS task

The experiment was performed on 4‐6‐year‐old male cynomolgus monkeys. The monkeys were maintained on 80% of their free‐feeding body weight throughout the experiment. Four monkeys were trained to perform the DMTS task by using the Cambridge Neuropsychological Test Automated Battery system (CeNes, Cambridge, UK).^35^ Briefly, a trial was initiated by the presentation of an image of a sample object on the screen. The monkey had to touch this object on the screen within 30 seconds. Subsequently, the sample object diminished from the screen, followed by a variable delay (0, 4, 8, or 16 seconds) before the reappearance of the sample object together with three other objects. The monkey had to choose the sample object from the four objects and a correct choice was rewarded by food. The ITI was 5 seconds and one session consisted of 96 trials (24 trials with delays of 0, 4, 8, and 16 seconds). The variable delay durations were randomly presented within the 96 trials. The criterion for the experimental use of monkeys was 70% or more of correct responses over the 96 trials. In the testing session, TAK‐137 (0.1 mg kg^−1^, p.o.) or corresponding vehicle was administered to monkeys 2 hours before DMTS testing. Ketamine (1.0 mg kg^−1^ i.m., as a salt) or corresponding vehicle was administered 15 minutes before DMTS testing. The administration followed a crossover design (n = 4). The dose of ketamine and the number of animals were determined on the basis of previous reports^23^, ^36^ and we confirmed that the ketamine‐induced deficits in the DMTS task was detectable under the experimental condition of n = 4. The correct responses were recorded for all the trials during the test sessions. The data were subdivided by delay interval, which consisted of 24 trials in each session, and are presented as the mean ± SEM of the percentage of correct trials out of the 96 trials per session.

### 2.9 Reversal learning test

The reversal learning test was conducted by b‐neuro^™^ (University of Manchester, UK). The training and testing methods have been previously described.^28^ All rats were tested in operant chambers with two‐lever Skinner boxes. The details of the apparatus were described in the previous report.^37^ The boxes were controlled by Med‐PC software (Version 2.0 for DOS or Med‐PC for Windows, Med Associates, Inc. Lafayette, IN). Programs were written using Medstate notation. At 12 weeks post weaning, the female Lister Hooded rats (n = 60) were initially trained to respond to food (45‐mg Noyes pellets, PJ Noyes Co Inc., Sandown Chemical Ltd) on a fixed ratio 1 (FR1) schedule of reinforcement in standard two‐lever Skinner boxes for 30 minutes. In the FR1 schedule, one press of either lever resulted in the delivery of a food pellet. The rats were food‐restricted to approximately 85% of free‐feeding body weight, maintained throughout the training and testing by feeding them approximately 12 g of standard lab chow per rat per day. Following a stable level of response to the FR1 schedule, the rats were trained to respond to food in the presence or absence of a visual cue in the form of a light stimulus above the lever. At the start of each session, the house light was turned on; both levers were introduced into the chamber, and the activation of one lever resulted in the delivery of a food pellet. One half of the animals were trained to respond in the presence of visual cue and the other half in the absence of the cue. Following a response on one lever, the house light was turned off and both levers were retracted for a period of 3 seconds; subsequently, the cycle was repeated. The rats were tested until 128 responses were obtained, or until the experimental session was terminated 30 minutes after the initiation of the training. The active lever was changed from session to session according to a pseudorandom Gellerman schedule. The rats participated in approximately 10‐15 sessions over 2‐3 weeks of training on the initial reward contingency and we ensured that they met the criterion on this initial task; that is, achieved at least 90% of correct responses with each lever active in at least two sessions. In the next step, they were trained on the opposite reward contingency, the reversal task. Once the criterion (90% of correct responses) was achieved on the reversal task, PCP (2 mg kg^−1^, as a salt) or vehicle was administered (i.p.) at a volume of 1 mL kg^−1^ twice per day for 7 days.^38^ During the PCP treatment period, the animals did not receive training to avoid association between drug treatment and cognitive performance. After 1‐week drug‐free period without training,^39^ the reversal testing was carried out on the animals. One day before the reversal testing, the rats were trained to respond to food using a randomly assigned contingency (ie, in the presence or absence of a cue). The session was terminated after the consumption of 128 pellets. Both PCP‐ and vehicle‐treated groups were required to achieve 90% of correct responses. If the animals failed to reach this criterion, they were subjected to further training until they were able to sustain 90% of the correct responses. The reversal testing consisted of two experimental phases: initial and reversal. In the initial phase, a consistent reward contingency like that of the previous training day was presented to the rats for 5 minutes or until the rats had earned 20 pellets. Following the initial phase, there was a time‐out period of 2 minutes (the house light was turned off). The animal stayed in the Skinner box during this time‐out period and the reversal phase was then initiated. In the reversal phase, the reward contingency was reversed so that the animals must respond in the opposite way from the initial test. The reversal test was also performed for 5 minutes. TAK‐137 (0.01, 0.1, or 1 mg kg^−1^, p.o.) or vehicle was administered 2 hours before the initial phase of the reversal testing. The numbers of animals used were as follows: n = 10 in the vehicle/vehicle‐treated group, n = 8 in the vehicle/PCP‐treated group, and n = 8 in the TAK‐137/PCP‐treated groups. The data are presented as the mean ± SEM of the percentage of correct responses in the initial and reversal phases of the reversal testing.

### 2.10 Statistical analysis

**Figure 1:**
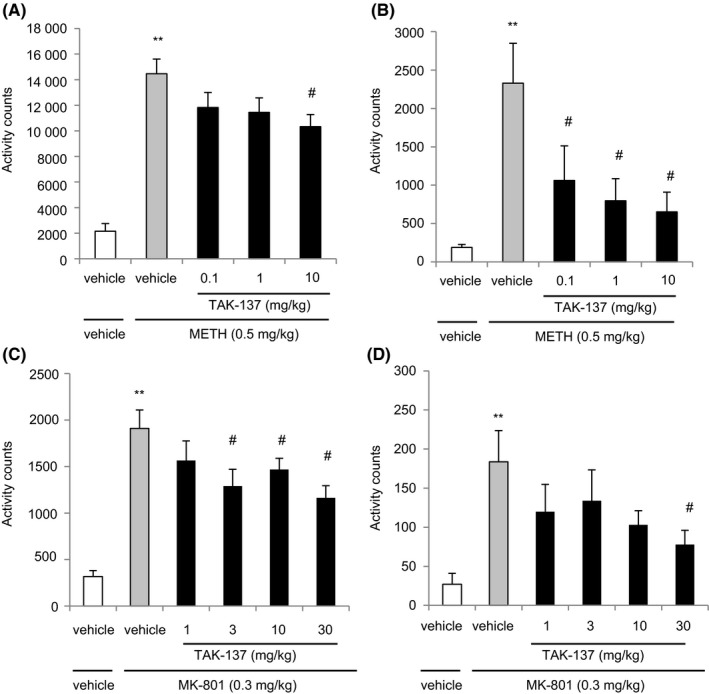
(A) The effect of TAK‐137 on METH‐induced hyperlocomotion in rats. The rats were acclimated in Plexiglas‐covered cages for at least 2 hours before the start of the experiment. TAK‐137 (0.1, 1, or 10 mg kg^−1^, p.o.) was administered 4 hours before METH administration (0.5 mg kg^−1^, s.c.). The data are presented as the mean ± SEM of cumulative locomotion for 0‐120 minutes. The numbers of animals (n) = 6 in the vehicle/vehicle‐treated group, n = 12 in the vehicle/METH‐treated group, n = 11 in the TAK‐137 (0.1 mg kg^−1^)/METH‐treated group, n = 13 in the TAK‐137 (1 mg kg^−1^)/METH‐treated group, and n = 13 in the TAK‐137 (10 mg kg^−1^)/METH‐treated group. **P < 0.01, statistically significant compared with the vehicle/vehicle‐treated group by Aspin‐Welch test, ^#^ *P* < 0.025, statistically significant compared with the vehicle/METH‐treated group by one‐tailed Williams’ test. (B) Data between 90 and 120 minutes after METH administration. **P < 0.01, statistically significant compared with the vehicle/vehicle‐treated group by Aspin‐Welch test. ^#^ *P* < 0.025, statistically significant compared with the vehicle/METH‐treated group by one‐tailed Williams’ test. (C) The effect of TAK‐137 on MK‐801‐induced hyperlocomotion in mice. The mice were acclimated in cages for at least 2 hours before the start of the experiments. TAK‐137 (1, 3, 10, or 30 mg kg^−1^, p.o.) was administered 2 hours before MK‐801 administration (0.3 mg kg^−1^, s.c.). The data are presented as the mean ± SEM of cumulative locomotion for 120 minutes after MK‐801 administration, n = 6 in the vehicle/vehicle‐treated group, n = 13 in the vehicle/MK‐801‐treated group, n = 14 in the TAK‐137 (1 mg kg^−1^)/MK‐801‐treated group, n = 13 in the TAK‐137 (3 mg kg^−1^)/MK‐801‐treated group, n = 16 in the TAK‐137 (10 mg kg^−1^)/MK‐801‐treated group, and n = 15 in the TAK‐137 (30 mg kg^−1^)/MK‐801‐treated group. **P < 0.01, statistically significant compared with the vehicle/vehicle‐treated group by Aspin‐Welch test; ^#^ *P* < 0.025, statistically significant compared with the vehicle/MK‐801‐treated group by one‐tailed Williams’ test. (D) Data between 90 and 120 minutes after MK‐801 administration. **P < 0.01, statistically significant compared with the vehicle/vehicle‐treated group by Aspin‐Welch test; ^#^ *P* < 0.025, statistically significant compared with the vehicle/MK‐801‐treated group by one‐tailed Shirley‐Williams’ test

**Figure 2:**
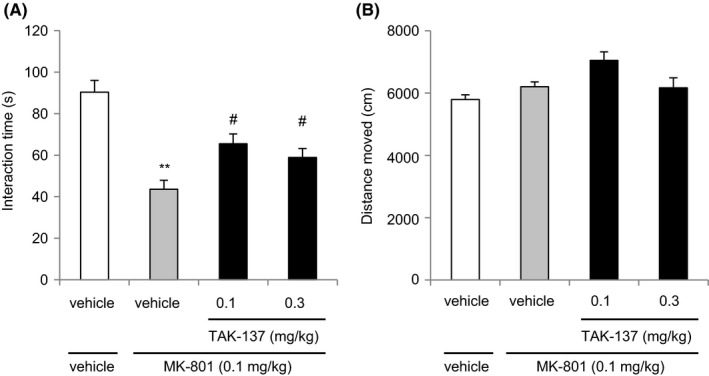
Effect of TAK‐137 on MK‐801‐induced deficits in the social behavior evaluated by the social interaction test in rats. (A) TAK‐137 (0.1 or 0.3 mg kg^−1^, p.o.) or corresponding vehicle, and MK‐801 (0.1 mg kg^−1^, s.c.) or corresponding vehicle were administered to rats 4 hours before testing. The interaction time of each treatment group is presented as the mean ± SEM (n = 14). **P < 0.01, statistically significant compared with the vehicle/vehicle‐treated group by Student's *t* test. ^#^ *P* < 0.025, statistically significant compared with the vehicle/MK‐801‐treated group by one‐tailed Williams’ test. (B) The distance traveled during testing over 10 minutes was measured and is presented as the mean ± SEM (n = 14)

**Figure 3:**
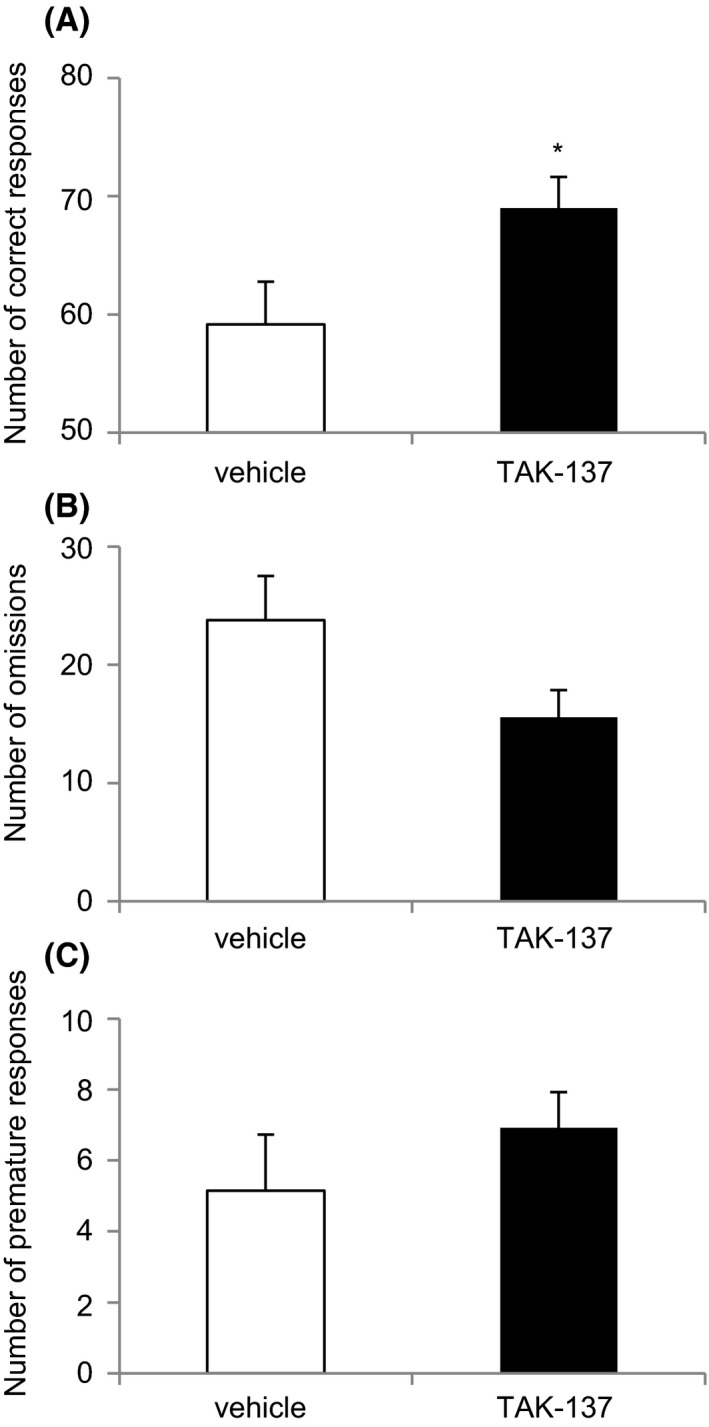
The effect of TAK‐137 on attention in the five‐choice serial reaction time task in rats. TAK‐137 (0.2 mg kg^−1^, p.o.) was administered 4 hours prior to the trial. (A) Correct responses are the total number of nose‐pokes in an illuminated aperture within the limited hold. (B) Omission responses are the number of nonresponses during the limited hold. (C) Premature responses represent the number of responses that occurred prior to stimulus presentation. The data are presented as the mean ± SEM (n = 13). Significant differences from the vehicle‐treated group are indicated by *P < 0.05 in the crossover ANOVA followed by a contrast test

**Figure 4:**
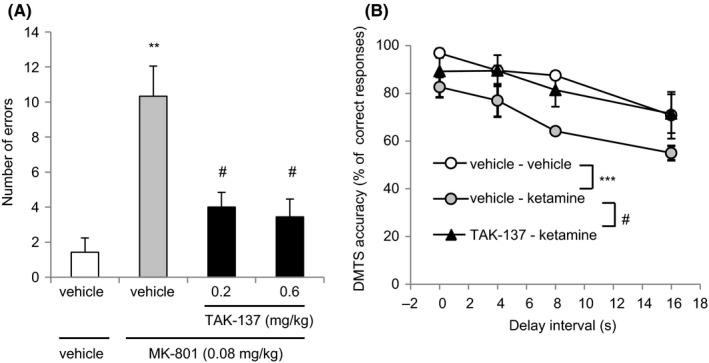
The effects of TAK‐137 on MK‐801‐ or ketamine‐induced deficits in working memory in the radial arm maze test in rats or delayed matching‐to‐sample task in monkeys. (A) TAK‐137 (0.2 or 0.6 mg kg^−1^, p.o.) or corresponding vehicle was administered to rats 2 hours before MK‐801 (0.08 mg kg^−1^, s.c.) or corresponding vehicle administration. The rats were placed on the maze 30 minutes after MK‐801 administration and entries into the arms were recorded. The data are presented as the mean ± SEM of the number of errors. The numbers of animals (n) = 7 in the vehicle/vehicle‐treated group, n = 9 in the vehicle/MK‐801‐treated group, n = 11 in the TAK‐137 (0.2 mg kg^−1^)/MK‐801‐treated group, and n = 9 in the TAK‐137 (0.6 mg kg^−1^)/MK‐801‐treated group. **P < 0.01, statistically significant compared between the vehicle/vehicle‐treated and the vehicle/MK‐801‐treated group by Aspin‐Welch test. ^#^ *P* < 0.025, statistically significant compared with the vehicle/MK‐801‐treated group by one‐tailed Williams’ test. (B) TAK‐137 (0.1 mg kg^−1^) or corresponding vehicle was orally administered 2 hours prior to ketamine or corresponding vehicle administration (1.0 mg kg^−1^, i.m.) in monkeys. The DMTS task was conducted 15 minutes after ketamine administration. Each plot represents the mean ± SEM of the percentage of correct responses from 96 trials per session (n = 4). The statistical analysis was performed using two‐way ANOVA followed by Bonferroni/Dunnet multiple comparisons, with significance set at ***P < 0.001 (the vehicle/vehicle‐treated group vs the vehicle/ketamine‐treated group) and at ^#^ *P* < 0.05 (the TAK‐137/ketamine‐treated group vs the vehicle/ketamine‐treated group)

**Figure 5:**
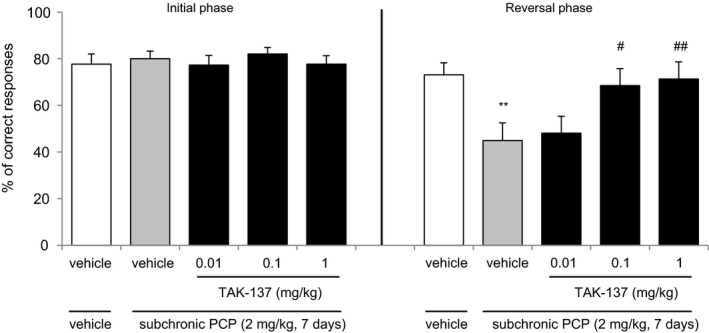
The effect of TAK‐137 on subchronic PCP‐induced deficits in cognitive flexibility in a reversal learning test in rats. The rats were trained to achieve 90% criterion of the correct response, followed by the administration of PCP (2 mg kg^−1^, i.p.) twice per day for 7 days without training. In the reversal testing, TAK‐137 (0.01, 0.1, or 1 mg kg^−1^, p.o.) or vehicle was administered 2 hours before the initial phase. The reversal phase was examined for 2 minutes immediately after the completion of the initial phase. The data are presented as the mean ± SEM of the percentage of correct responses in the initial and reversal phases. The numbers of animals (n) = 10 in the vehicle/vehicle‐treated group, n = 8 in the vehicle/PCP‐treated group, and n = 8 in the TAK‐137/PCP‐treated groups. **P < 0.01, statistically significant compared with the vehicle/PCP‐treated group by Student's *t* test. ^#^ *P* < 0.05, ^##^ *P* < 0.01, statistically significant compared with the vehicle/PCP‐treated group by one‐way ANOVA followed by LSD test

The data are presented as the mean ± SEM Statistical analysis was performed by EXSUS (CAC Croit Corporation). The *F* test, followed by Student's *t* test (for data with homoscedasticity with *P *≥* *0.2 by *F* test) or Aspin‐Welch test (for data with heteroscedasticity with *P* < 0.2 by *F* test) with multiplicity adjustment by Bonferroni correction were used for comparisons between two groups: vehicle/vehicle and vehicle/METH (Figure 1A and B), vehicle/MK‐801 (Figure 1C and D), and vehicle/PCP treatments (Figure 5). The level of significance in each *t* test was designated by the values of *P* < 0.05. Bartlett's test was performed to test the equality of variances, followed by a one‐tailed Williams’ test (for parametric data, *P *≥* *0.05 by Bartlett's test) or one‐tailed Shirley‐Williams’ test (for nonparametric data, *P* < 0.05 by Bartlett's test) to assess the dose‐dependent effects of TAK‐137 compared with the vehicle/METH‐ or vehicle/MK‐801‐treatment groups (Figures 1 and 2) and differences were considered significant for *P* values of <0.025. The analysis of variance (ANOVA) followed by post hoc analysis of contrast test (5‐CSRTT, Figure 3), Bonferroni/Dunnett multiple comparisons (DMTS task, Figure 4B), or a least significant difference (LSD) test (reversal learning test, Figure 5), which was used for crossover or repeated design experiments. The effect of TAK‐137 was compared with vehicle‐ (Figure 3), vehicle/ketamine‐ (Figure 4), or vehicle/PCP‐treatment groups and the differences were considered significant for *P* values <0.05.

## 3 RESULTS

### 3.1 Effect of TAK‐137 on the positive symptoms of schizophrenia

The total count of locomotion over 120 minutes after METH administration in the METH‐treated group (14470 ± 1136 counts) was significantly higher than that in the vehicle‐treated group (2154 ± 588.7 counts) (*P* = 0.0394, *F* test followed by *P* < 0.01, Aspin‐Welch test; Figure 1A). TAK‐137 significantly decreased the METH‐induced hyperlocomotion to 10 328 ± 943.5 counts in the TAK‐137 (10 mg kg^−1^)/METH group (*P* = 0.9298, Bartlett's test followed by *P* < 0.025, one‐tailed Williams’ test, compared with the vehicle/METH‐treated group). The efficacy of TAK‐137 was most prominent between 90 and 120 minutes after METH administration. TAK‐137 at 0.1, 1, and 10 mg kg^−1^ reduced the total counts of locomotion in the TAK‐137/METH‐treated group to 1062 ± 449.7, 797.0 ± 287.9, and 653.4 ± 254.8, respectively in comparison with that in the vehicle/METH‐treated group (2328 ± 521.3 counts) (*P* = 0.0917 in Bartlett's test followed by *P* < 0.025, one‐tailed Williams’ test; Figure 1B).

The effect of TAK‐137 on hyperlocomotion induced by MK‐801 (0.3 mg kg^−1^ as a salt, s.c.) was also investigated in mice (Figure 1C). The total count of locomotion over 120 minutes after MK‐801 administration in the MK‐801‐treated group (1910 ± 197.8 counts) was significantly higher than that in the vehicle‐treated group (317.7 ± 63.67 counts) (*P* = 0.0035, *F* test followed by *P* < 0.01, Aspin‐Welch test; Figure 1C). TAK‐137 (3, 10, and 30 mg kg^−1^) significantly decreased MK‐801‐induced hyperlocomotion to 1290 ± 180.3, 1469 ± 119.0, and 1164 ± 128.8 counts, respectively in the TAK‐137/MK‐801‐treated group in comparison with that in the vehicle/MK‐801‐treated group (1910 ± 197.8 counts) (*P* = 0.2894 in Bartlett's test followed by *P* < 0.025, one‐tailed Williams’ test). In contrast to the result of METH‐induced hyperlocomotion, TAK‐137 did not induce prominent effects on locomotion between 90 and 120 minutes after MK‐801 administration; a significant effect was observed only at 30 mg kg^−1^ (*P* = 0.0131 in Bartlett's test followed by *P* < 0.025, one‐tailed Shirley‐Williams’ test; Figure 1D).

Unlike reported antipsychotics with dopamine D2 antagonism, TAK‐137 did not induce a significant cataleptic response and increase of prolactin and glucose levels in plasma (Figure S1).

### 3.2 Effect of TAK‐137 on the negative symptoms of schizophrenia

#### 3.2.1 Social behavior

The interaction time in the vehicle/MK‐801 (0.1 mg kg^−1^, as a salt)‐treated group (43.6 ± 4.33 seconds) was significantly lower than that in the vehicle/vehicle‐treated group (90.33 ± 5.71 seconds, *P* = 0.3285, *F* test followed by *P* < 0.01, Student's *t* test; Figure 2A). The interaction time was significantly increased by the administration of 0.1 and 0.3 mg kg^−1^ TAK‐137 to 65.5 ± 4.78 and 58.9 ± 4.30 seconds, respectively (*P* = 0.9146 in Bartlett's test followed by *P* < 0.025, one‐tailed Williams’ test; Figure 2A). MK‐801 administration did not affect the total distance moved. The total distances moved, in the presence and absence of MK‐801 were 5796 ± 149 cm and 6205 ± 155 cm, respectively. TAK‐137 treatment did not significantly increase the distance moved in the presence of MK‐801 (7048 ± 276 cm and 6171 ± 324 cm at 0.1 and 0.3 mg kg^−1^ with MK‐801, respectively) (Figure 2B).

### 3.3 Effects of TAK‐137 on the cognitive symptoms of schizophrenia

The plasma and brain concentrations of TAK‐137 under fasted conditions were 67% and 71% of those under the fed conditions (Table S1). Thus, 0.2 and/or 0.6 mg kg^−1^ of TAK‐137 were used in the experiments requiring restricted food consumption.

#### 3.3.1 Attention

The vehicle‐treated group achieved 59.1 ± 3.63 correct responses in 100 trials; this was significantly increased by TAK‐137 (0.2 mg kg^−1^, p.o.) to 69.0 ± 2.61 responses in 100 trials (*F*(1, 11) = 5.28, *P* < 0.05, cross over ANOVA; Figure 3A). There was no significant difference in the number of omissions and premature responses between the vehicle‐treated group and the TAK‐137‐treated group. The number of omission responses recorded was 23.8 ± 3.73 and 15.5 ± 2.54 in the vehicle‐treated group and the TAK‐137‐treated group, respectively (*F*(1, 11) = 4.56, cross over ANOVA, *P* = 0.056; Figure 3B). The number of premature responses (a response prior to light stimulation) was 5.15 ± 1.57 and 6.92 ± 1.00 in the vehicle‐treated group and the TAK‐137‐treated group, respectively (Figure 3C).

#### 3.3.2 Working memory

In the testing session of the RAM test, treatment with MK‐801 (0.08 mg kg^−1^, as a salt, s.c.) disrupted the performance of the well‐trained rats in the collection of the pellets on the maze and increased the number of errors from 1.43 ± 0.81 to 10.33 ± 1.72 (*P* = 0.0461, *F* test followed by *P* < 0.001, Aspin‐Welch test, compared with the vehicle‐treated group; Figure 4A). TAK‐137 (0.2 and 0.6 mg kg^−1^) significantly reduced the number of errors to 4.00 ± 0.85 and 3.44 ± 1.02, respectively (*P* = 0.1488 in Bartlett's test followed by *P* < 0.025, one‐tailed Williams’ test, compared with the vehicle/MK‐801‐treated group; Figure 4A).

In the DMTS task, the monkeys were trained to correctly identify a sample object from four objects projected on the monitor after various delay intervals of 0 (no delay), 4, 8, and 16 seconds. Ketamine treatment significantly decreased the percentage of correct responses compared with vehicle treatment, with values of (96.88 ± 1.99) % and (82.63 ± 4.29) % recorded with 0 second interval, (89.58 ± 2.08) % and (76.99 ± 6.82) % recorded with 4 seconds interval, (87.50 ± 1.70) % and (64.18 ± 1.48) % recorded with 8 seconds interval, and (70.8 ± 9.77) % and (55.0 ± 3.00) % recorded with 16 seconds interval after the administration of vehicle and ketamine, respectively (*F*(1, 24) = 18.39, *P* < 0.001, two‐way ANOVA; Figure 4B). The plasma concentration of TAK‐137 at 0.1 mg kg^−1^ in monkeys (40 ng mL^−1^ as *C*_max_) was comparable to that at 0.1 mg kg^−1^ (25 ng mL^−1^ as *C*_max_)—the dose which showed pharmacological efficacies, in rats.^23^ At 0.1 mg kg^−1^, TAK‐137 significantly ameliorated ketamine‐induced cognitive deficits, with percentage accuracy of (89.3 ± 6.51) %, (89.5 ± 6.54) %, (81.4 ± 6.97) %, and (71.5 ± 8.16) % at 0, 4, 8, and 16 seconds interval memory test, respectively (*F*(1, 24) = 7.29, *P* < 0.05, two‐way ANOVA; Figure 4B).

#### 3.3.3 Cognitive flexibility

We evaluated the effects of TAK‐137 on subchronic PCP‐induced deficits in a reversal learning task. In the initial phase of the testing session, there were no significant differences in the percentage of correct responses in all the groups: (77.7 ± 4.30) % in the vehicle/vehicle‐treated group, (80.0 ± 3.19) % in the vehicle/PCP‐treated group (*P* = 0.68, Student's *t* test, compared with the vehicle/vehicle‐treated group), (77.3 ± 4.07) % in the TAK‐137 (0.01 mg kg^−1^)/PCP‐treated group, (82.0 ± 2.73) % in the TAK‐137 (0.1 mg kg^−1^)/PCP‐treated group, and (77.7 ± 3.60) % in the TAK‐137 (1 mg kg^−1^)/PCP‐treated group (*F*(3, 28) = 0.3992, *P* = 0.75, one‐way ANOVA; Figure 5). In the reversal phase, the percentage of correct responses was significantly lower in the vehicle/PCP‐treated group than in the vehicle‐treated group ((44.9 ± 7.63) % vs (73.1 ± 5.20) %) (*P* = 0.4394, *F* test followed by *P* < 0.01, Student's *t* test; Figure 5). TAK‐137 significantly ameliorated the PCP‐induced deficits and increased the percentage of correct responses to (68.5 ± 7.28) % at 0.1 mg kg^−1^ and (71.3 ± 7.34) % at 1 mg kg^−1^ (*F*(3, 28) = 3.41, *P* < 0.05 and *P* < 0.01, at 0.1 and 1 mg kg^−1^, respectively, one‐way ANOVA).

### 3.4 Effects of TAK‐137 under the combination with olanzapine

We studied the effects of the combination of olanzapine (3 mg kg^−1^), one of the antipsychotics, and TAK‐137 (0.1, 1, and 10 mg kg^−1^) on METH‐induced hyperlocomotion, cataleptic response, and plasma prolactin level (Figure S2). The results showed that the inhibitory effect of olanzapine on METH‐induced hyperlocomotion was not affected by TAK‐137 (Figure S2A). TAK‐137 did not exacerbate the cataleptic response and plasma prolactin level (Figure S2B and C, respectively). In addition, co‐treatment of TAK‐137 (1 mg kg^−1^, p.o.) and olanzapine (3 mg/kg, p.o.) did not affect the effect on cognitive improvement in novel object recognition test in rats (Figure S2D).

## 4 DISCUSSION

Schizophrenia is a chronic psychiatric disorder with a spectrum of symptoms: positive, negative, and cognitive symptoms. Several hypotheses for the pathophysiology of schizophrenia have been indicated based on clinical findings.^40^ The hyperdopamine hypothesis is considered to indicate the main cause of positive symptoms, and the hypoglutamate hypothesis has been proposed not only for positive symptoms but also for negative and cognitive symptoms.^41^ Recent genetic findings in genome‐wide association studies revealed that genes related to the NMDA receptor signaling complex are associated with an increased risk of schizophrenia.^42^ Moreover, NMDA receptor antagonists are reported to induce schizophrenia‐like psychosis, social dysfunction, and cognitive impairments in healthy volunteers, and exacerbate symptoms in patients with schizophrenia.^43^, ^44^, ^45^ Indeed, several small molecules that enhance NMDA signaling, such as D‐serine and a GlyT1 inhibitor, have been reported to improve negative and cognitive function in clinical trials.^13^, ^14^ Thus, modulation of NMDA function is likely a promising approach for the treatment of patients with schizophrenia.

In the hypoglutamate state, NMDA receptor signaling on the parvalbumin (PV)‐positive GABA interneurons is decreased in the cortex and hippocampus.^46^ PV‐positive GABA interneurons play a key role in cognitive function through the production of neural oscillation, especially gamma oscillation.^47^ Moreover, a significantly low level of gamma oscillation during cognitive tasks is reported in patients with schizophrenia.^48^ Thus, the clinical characterization of gamma oscillation could be a promising biomarker.

Schizophrenia is recognized as a neurodevelopmental disease and altered fetal or neonatal environments, such as viral infection, are indicated to be risk factors.^49^ Methylazoxymethanol acetate (MAM) exposure^50^ and polyriboinosinic‐polyribocytidylic acid (Poly I:C) injection^51^ have been reported to mimic maternal mitotoxin or viral exposure during pregnancy in rodents and these manipulations result in the hypofunctioning of NMDA signaling in PV‐positive GABA interneurons.^52^ These preclinical results further support the association of the hypoglutamate state with the pathophysiology of schizophrenia. To evaluate the effects of TAK‐137 on glutamate hypofunction, we used both acute and sub‐chronic treatments with NMDA receptor antagonists. The acute treatment of MK‐801 and ketamine can inhibit NMDA receptor signaling in PV‐positive neurons, and the sub‐chronic treatment of PCP is reported to reduce PV expression.^9^

The activation of the AMPA receptor leads to increased NMDA receptor function; thus, AMPA receptor potentiators have potential as therapeutic drugs for schizophrenia. However, risks such as the bell‐shaped response and the induction of seizures have been indicated in previously reported compounds. The in vivo studies of LY451646 and LY404187 revealed their steeply bell‐shaped responses. For example, c‐fos induction in rats was detected only at 0.5 mg kg^−1^ after 0.05, 0.5, and 5 mg kg^−1^ treatments.^53^ BDNF mRNA in the rat hippocampus was induced only at 0.5 mg kg^−1^ after 7‐day dosing at 0.125, 0.25, 0.5, and 1 mg kg^−1^.^54^ Such a bell‐shaped response makes it difficult to select the optimal dosage in clinical studies given the heterogeneity of drug metabolizing enzymes in humans. In addition, we reported that LY451646 showed narrow safety margins between exposure at the effective dose in rat novel object recognition test (NORT) and at the maximum dose in the absence of seizures, that is, 3.1‐ and 7.5‐fold in the AUC_brain_ and brain *C*_max_, respectively.^23^ Thus, it can be presumed that the doses of AMPA receptor potentiators in previous clinical studies could not be increased to the exposure level required for efficacy owing to their narrow safety margin against seizure. Therefore, compounds with improved bell‐shaped responses and reduced seizure risk should be generated.

We revealed that the bell‐shaped response was related to the agonistic property of compounds, which was detected only in primary cells and not in recombinant cells.^22^ Thus, the agonistic property of each compound should be characterized using the respective physiological receptor, although such complex conditions may not be appropriate for a high throughput screening (HTS) assay. Therefore, we established an original screening strategy, which included a unique binding assay for HTS, and identified TAK‐137.^23^ In the DMTS test in naive monkeys, TAK‐137 enhanced cognitive performance at 0.03, 0.1, and 1 mg kg^−1^, whereas LY451656 enhanced performance only at 0.1 mg kg^−1^ when dosed at 0.03, 0.1, and 1 mg kg^−1^. The safety margins between the exposure yielding cognitive enhancement in NORT and the maximum exposure in the absence of seizure were 116‐ and 43.7‐fold in the AUC_brain_ and brain *C*_max_, respectively. Therefore, TAK‐137 is superior to LY451646 in terms of the bell‐shaped responses and the safety margin.

In this study, we investigated effects of TAK‐137, a compound with low agonistic properties, on animal models of schizophrenia. At 10 mg kg^−1^, TAK‐137 significantly inhibited METH‐induced hyperlocomotion in rats to 33.6 ± 7.66% of that in the control (Figure 1A and B); however, the percentage of inhibition was lower than that of antipsychotics, which exhibited greater than 50% inhibition under the 60% occupancy of D2R.^26^, ^55^ TAK‐137 may therefore have limited efficacy against the positive symptoms of schizophrenia. Negative symptoms are rarely improved by current antipsychotics.^56^ In this study, TAK‐137 at 0.1 and 0.3 mg kg^−1^ significantly ameliorated MK‐801‐induced deficits in SI (Figure 2A) without a significant increase in locomotion (Figure 2B). This indicated that the effect was not a secondary effect of an increase in locomotion and that TAK‐137 has the potential to improve the social behavior associated with NMDA receptor hypofunction. Further characterization should be considered using other in vivo tests, such as the sucrose preference test, as several neuronal networks may be related to the negative symptoms.^2^, ^57^, ^58^ The effects of TAK‐137 on cognitive symptoms, especially attention, working memory, and executive control, were investigated as these were recognized as the key cognitive domains impaired in schizophrenia by the Measurement and Treatment Research to Improve Cognition in Schizophrenia initiative.^59^, ^60^ The 5‐CSRTT contains aspects of the continuous performance task in humans,^61^ the RAM test in rats can be adapted to mimic the N‐back task in humans,^62^ and the monkey DMTS task can be translated to the human DMTS task.^63^ A reversal learning task is regarded as the evaluation of the cognitive flexibility required for rule generation and selection.^64^, ^65^ TAK‐137 improved these cognitive dimensions in animal models with acute or sub‐chronic NMDA receptor antagonists (Figures 3, 4, 5, indicating that it may be effective for the treatment of multiple cognitive domains in patients with schizophrenia. Recently, an AMPA receptor potentiator, PF‐04958242, was reported to significantly reduce ketamine‐induced impairment in immediate recall and working memory tasks in healthy human subjects at plasma concentrations similar to that in nonhuman primates.^66^ The data further supported that AMPA receptor activation can counteract the hypoglutamate state induced by NMDA inhibitors in humans.

The positive symptoms of schizophrenia are well‐controlled by current antipsychotics compared with other symptoms. Considering the reported findings and the results in this study, TAK‐137 may be characterized by particular efficacy against the negative and cognitive symptoms. Therefore, we investigated the impact of the concomitant use of TAK‐137 and an antipsychotic (Figure S2). TAK‐137 did not interfere with the effects of olanzapine on METH‐induced hyperlocomotion and did not exacerbate the cataleptic response and the plasma prolactin level. In addition, co‐treatment of olanzapine did not alter the effects of TAK‐137 on cognition. Thus, the combination of TAK‐137 with the currently available antipsychotics may be beneficial in treating multiple symptoms of schizophrenia. A synergistic enhancement of efficacies was not detected in this study, which may be a result of the different mechanisms of action of D2R antagonism by olanzapine and enhancement of the glutamate signal by TAK‐137.

Another critical issue in the development of drugs for schizophrenia is the heterogeneity of the disease etiology and biology of schizophrenia. Indeed, the importance of patient segmentation by biophenotype has been suggested.^67^ Patients in the hypoglutamate state may be determined, for example, by mismatch negativity or gamma oscillations.^68^, ^69^ The proof of concept should be investigated in the clinical studies of patients selected by such biomarkers.

In conclusion, TAK‐137, an AMPA receptor potentiator with a low agonistic activity, broader effective dose range, and greater safety margin against seizure, was shown to be efficacious in various animal models of schizophrenia. Thus, TAK‐137‐type AMPA receptor potentiators may be promising therapeutic options in neuropsychiatry and neurological diseases.

## DISCLOSURES

This work was funded by Takeda Pharmaceutical Company Limited.

## AUTHOR CONTRIBUTIONS

All authors are employees of Takeda Pharmaceutical Company Limited.

Participated in research design: Tanaka, Kunugi, A. Suzuki, and Kimura.

Conducted experiments: Tanaka, Kunugi, A. Suzuki, N. Suzuki, and M. Suzuki.

Performed data analysis: Tanaka, Kunugi, A. Suzuki, N. Suzuki, M. Suzuki, and Kimura.

Wrote or contributed to the writing of the manuscript: Tanaka, and Kimura.
