# Supplementary figures and images for: Preclinical characterization of AMPA receptor potentiator TAK‐137 as a therapeutic drug for schizophrenia
Source: Pharmacol Res Perspect. 2019 May 9;7(3):e00479. doi: 10.1002/prp2.479 (PMC6507438; doi:10.1002/prp2.479)

Supplementary Fig. 1

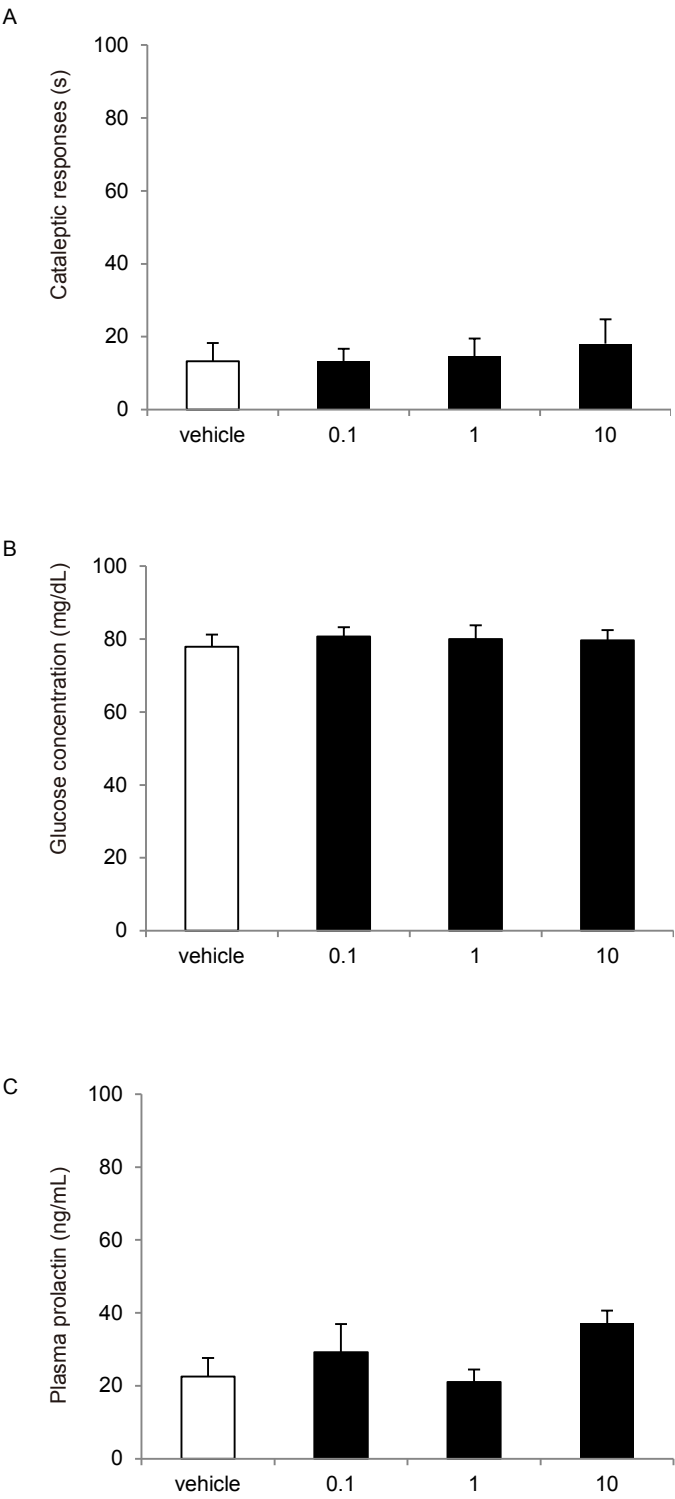

Supplementary Fig. 2

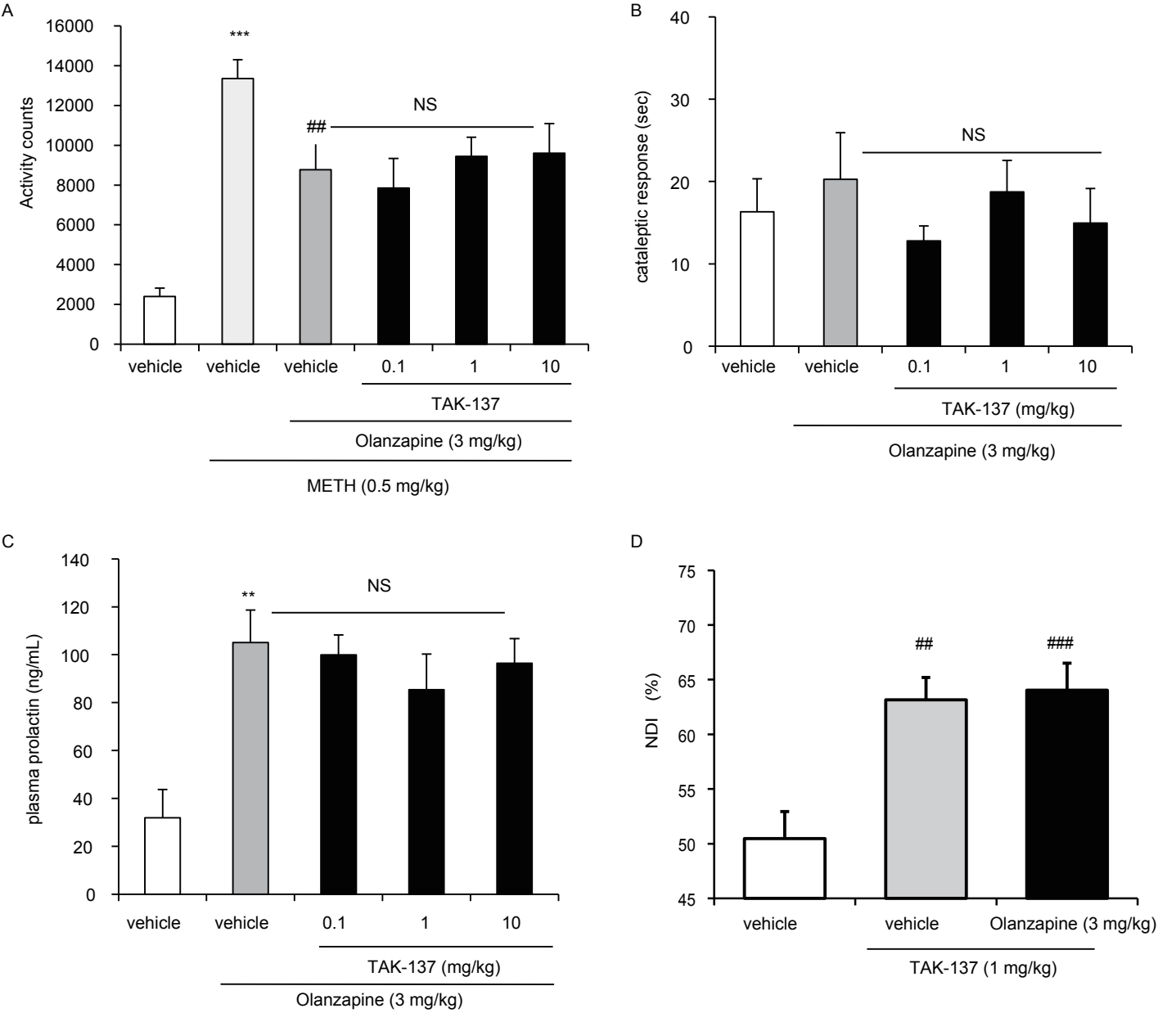

Supplement: Supplementary file 1 [file PRP2-7-e00479-s001.pdf]
