# Supplementary material for: Preclinical characterization of AMPA receptor potentiator TAK‐137 as a therapeutic drug for schizophrenia
Source: Pharmacol Res Perspect. 2019 May 9;7(3):e00479. doi: 10.1002/prp2.479 (PMC6507438; doi:10.1002/prp2.479)
Supplement: Supplementary file 2 [file PRP2-7-e00479-s002.docx]

**Supplementary Materials and Methods**

Novel Object Recognition **Test (NORT)**

On day 1, male Long-Evans rats at 6 weeks old (Japan SLC Inc., Hamamatsu, Japan) were allowed to habituate to the behavioral test room environment for over 1 h, and then they were allowed to habituate to the empty test box [a gray-colored polyvinyl chloride box (40 × 40 × 50 cm)] for 10 min individually. Testing was composed of two 3-min trials, called the acquisition and the retention trials. These trials were separated by a 48-h inter-trial interval (ITI). On day 2, during the acquisition trial, rats were allowed to explore two identical objects (A1 and A2) for 3 min. On day 4, in the retention trial, rats were allowed to explore a familiar object (A3) and a novel object (B) for 3 min. Exploration of an object was defined as licking or touching the object with forelimbs while sniffing. Leaning against the object to look upward, and standing, or sitting on the object were excluded. The exploration time for each object (A1, A2, A3, and B) in each trial was scored manually. A novelty discrimination index (NDI) was calculated as the novel object interaction time / total interaction time × 100 (%).

**Measurement of cataleptic response**

The experimental procedure was performed in accordance with the method reported by Hoffman and Donovan ([Hoffman and Donovan, 1995](#_ENREF_39)). On the day before the experiment, the rats were trained to grab a horizontal metal bar at a 13 cm height with their forelimbs for approximately 30 s. Catalepsy-like behavior was measured 4 h after the administration of vehicle or TAK-137 (0.1, 1, and 10 mg kg^-1^, p.o.) in blinded condition (n = 12). A rat was placed in front of the metal bar, with its forelimbs placed on the bar, and the time until the rat removed both forelimbs from the bar was recorded. Animals with sustained grabbing posture for 90 s were removed from the apparatus and assigned a latency time of 90 s. The average of three consecutive trials was recorded to determine the duration of the cataleptic response. The data are presented as the mean ± S.E.M. of the average latency time before the removal of both forelimbs from the bar in three consecutive trials.

**Measurement of plasma prolactin levels**

TAK-137 (0.1, 1, and 10 mg kg^-1^, p.o.) was administered 4 h before decapitation and whole blood samples were collected (n = 6). The collected samples were stored on ice in EDTA-containing tubes. Plasma was obtained by the centrifugation of whole blood (4 °C, 12000 rpm, and 15 min). The plasma supernatant was collected, transferred to another tube, and stored in a deep-freezer until use. The plasma concentrations of prolactin were measured by using an enzyme-linked immunoassay kit (Bertin Pharma, Montigny le Bretonneux, France).

**Measurement of plasma or brain concentration of compounds**

Male Sprague-Dawley rats (Charles River Laboratories Japan) were used in this experiment. The rats were administered TAK-137 (0.1 mg kg^-1^, p.o.), and were sacrificed by decapitation after 2 h for the collection of blood and brain tissues. The plasma was separated from the blood samples by centrifugation. The brain tissue was homogenized with saline. The concentrations of TAK-137 in the plasma and brain were determined by using liquid chromatography-tandem mass spectrometry.

**Combination effects of olanzapine and TAK-137 in locomotion, NORT, cataleptic response, and plasma prolactin levels**

Olanzapine was extracted from Zyprexa® (Eli Lilly and Company, Indianapolis, Indiana, US) at KNC Laboratories Co., Ltd. (Kobe, Japan) and dissolved in 1.5 % (v/v) lactic acid. The pH of the solution was adjusted to neutral using 1 M NaOH. Olanzapine was administered (p.o.) at a volume of 2 mL kg^-1^ in rats. After the habituation, TAK-137 (0.1, 1, and 10 mg kg^-1^, p.o.), olanzapine, or corresponding vehicle was orally administered 4 h (TAK-137) or 1 h (olanzapine) before METH administration (0.5 mg kg^-1^, s.c.). In NORT, TAK-137 (1 mg kg^-1^) and olanzapine (3 mg kg^-1^) were orally administered 2 h or 1 h prior to the acquisition and the retention trials, respectively. (n = 10). Catalepsy-like behavior was measured 4 h after the administration of vehicle, TAK-137 (0.1, 1, and 10 mg kg^-1^, p.o.), or olanzapine (3 mg kg^-1^, p.o.) in blinded condition (n =12). For the measurement of plasma prolactin levels, TAK-137 (0.1, 1, and 10 mg kg^-1^, p.o.) was administered 4 h before, and olanzapine (3 mg kg^-1^, p.o.) was administered 1.5 h before decapitation to collect whole blood (n = 6).

**Statistics**

Statistical analysis was performed by EXSUS (CAC Croit Corporation). Single measurement type statistical analysis was performed for the results of the locomotion test. In each test, the effect of METH was analyzed by t test to compare between two groups: vehicle/vehicle treatment and vehicle/METH treatment. Prior to the t test, F test was performed to decide parametric or non-parametric. Based on the F test, Student's t test for equal variance (*p* ≥ 0.2 by F test) or Aspin–Welch test for unequal variance (*p <* 0.2 by F test) was used. The level of significance in each t test was designated by the values of *p* < 0.05 through multiplicity adjustment by Bonferroni correction. The effect of olanzapine (3 mg kg^-1^ ) was also compared with vehicle/METH-treated group by F test followed by Student's t test. In the experiments that examined the effects of multiple doses of test compounds, statistical significance was analyzed using Bartlett’s test to test for the homogeneity of variances, followed by one-tailed Williams’ test (for parametric data, *p* > 0.05 by Bartlett’s test) or one-tailed Shirley-Williams’ test (for non-parametric data, *p* ≤ 0.05 by Bartlett’s test) for the comparison of the dose-dependent effects of the multiple doses of the test compounds with the vehicle group. Differences at *p* values < 0.025 were considered statistically significant. In the NORT, the effect of combination of TAK-137 and olanzapine was assessed by using two-way ANOVA followed by Bonferroni/Dunnet multiple comparisons with significance set at *p* < 0.05.

**References in Supplementary Information**

Hoffman, D. C. and Donovan, H. (1995) Catalepsy as a rodent model for detecting antipsychotic drugs with extrapyramidal side effect liability. *Psychopharmacology* **120**:128-133.

**Supplementary Table**

Table S1

Pharmacokinetic profile of TAK-137 in rats under non-fasted and fasted condition. TAK-137 at 0.1 mg kg^-1^ was orally administered to rats (n = 3).


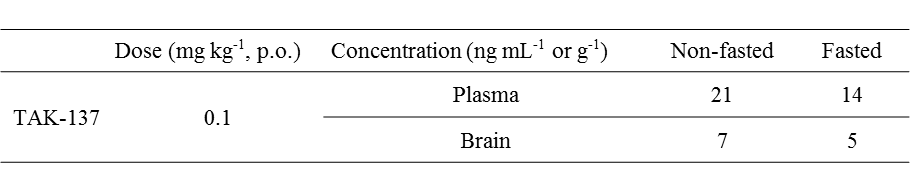


**Supplementary Figure Legends**

Supplementary Fig. 1. The effects of TAK-137 on cataleptic response, and plasma glucose and prolactin level in rats. (A) TAK-137 (0.1, 1, or 10 mg kg^-1^) was administered 4 h before test. The duration of grabbing the bar was measured to evaluate cataleptic response and is presented as mean ± S.E.M. (n = 6). (B) TAK-137 (0.1, 1, or 10 mg kg^-1^) was administered 4 h before blood collection. Data are presented as mean ± S.E.M. of plasma prolactin levels (n = 5). (C) TAK-137 (0.1, 1, or 10 mg kg^-1^) was administered 4 h before blood collection. Data are presented as mean ± S.E.M. of plasma prolactin levels (n = 6-7).

Supplementary Fig. 2. Effects of TAK-137 on the pharmacological effects and side effects of olanzapine in METH-induced hyperlocomotion, NORT, cataleptic response, and plasma prolactin level in rats. (A) TAK-137 (0.1, 1, or 10 mg kg^-1^, p.o.) and olanzapine (3 mg kg^-1^, p.o.) was administered 4 h and 1 h respectively, before METH administration (0.5 mg kg^-1^, s.c.). Total locomotor activities for 120 min after METH administration are presented as mean ± S.E.M. The numbers of animals used were 6, 12, 13, 8, and 9 in the vehicle-treated group, vehicle/METH-treated group, olanzapine/METH-treated group, olanzapine/TAK-137 (0.1 or 1 mg kg^-1^)–METH-treated group, and olanzapine/TAK-137 (10 mg kg^-1^)/METH-treated group respectively. ****p* < 0.001, statistically significant compared with vehicle-treated group by Aspin–Welch test. ##*p* < 0.01, statistically significant compared with vehicle/METH-treated group by Aspin–Welch test. NS, not significant (versus olanzapine/METH-treated group; one-tailed Williams’ test). (B) TAK-137 (0.1, 1, or 10 mg kg^-1^) and olanzapine (3 mg kg^-1^) were administered 4 h before testing. The duration of grabbing the bar was measured to evaluate cataleptic response and is presented as mean ± S.E.M. (n = 12). NS, not significant (versus vehicle-olanzapine group; one-tailed Williams’ test). (C) TAK-137 (0.1, 1, or 10 mg kg^-1^) was administered 4 h, and olanzapine (3 mg kg^-1^) was administered 1.5 h before blood collection. Data are presented as mean ± S.E.M. of plasma prolactin levels (n = 6). ***p* < 0.01, statistically significant compared with vehicle-treated group by Aspin–Welch test. NS, not significant (versus vehicle-olanzapine- treated group; one-tailed Williams’ test). (B) TAK-137 (1 mg kg^-1^) and olanzapine (3 mg kg^-1^) were orally administered 2 h or 1 h prior to the acquisition and the retention trials, respectively (n = 10). A novelty discrimination index (NDI) was calculated as the novel object interaction time / total interaction time × 100 (%) and are presented as mean ± S.E.M.. ##*p* < 0.01, ###*p* < 0.001 statistically significant compared with vehicle-treated group by ANOVA followed by Bonferroni/Dunnet multiple comparisons.
